# Licoflavone B Suppresses Influenza A Virus by Targeting the Viral RNA-Dependent RNA Polymerase (RdRp)

**DOI:** 10.3390/v17091157

**Published:** 2025-08-24

**Authors:** Pu Fan, Peng Lv, Sen Zhang, Zheng Zhu, Kewen Qian, Jin Han, Yue Cui, Ye Feng, Zeya Li, Li Qiang, Yunzhu Dong, Ting Fang, Tao Jiang, Changming Yu, Xiangyang Chi

**Affiliations:** 1 School of Pharmacy, Nanjing University of Chinese Medicine, Nanjing 210023, China; fanpu0518@163.com; 2 Academy of Military Medical Sciences, Beijing 100071, China; lp1205@foxmail.com (P.L.); zhuzheng0627@163.com (Z.Z.); qiankewen1205@163.com (K.Q.); harncy@126.com (J.H.); cuiyue0035@163.com (Y.C.); 18610356616@163.com (Z.L.); qiangliupup@163.com (L.Q.); 13811429044@163.com (Y.D.); 18612006386@163.com (T.F.); 3 State Key Laboratory of Pathogen and Biosecurity, Academy of Military Medical Sciences, Beijing 100071, China; zhangsen2260@163.com (S.Z.); fengye621@126.com (Y.F.)

**Keywords:** influenza A virus (IAV), antiviral treatment, licoflavone B, RNA-dependent RNA polymerase (RdRp)

## Abstract

Influenza A virus pandemics pose a persistent global health threat, and emerging antiviral resistance underscores the critical importance of developing novel broad-spectrum therapeutic agents. Building on licorice’s (*Glycyrrhiza* spp.) historical use in traditional Chinese medicine for respiratory infections—as documented in the Chinese Guidelines for Diagnosis and Treatment of Influenza—and its demonstrated anti-SARS-CoV-2 activity, we identified licoflavone B as a potent anti-influenza agent, bridging ethnopharmacological knowledge with mechanistic validation. In this study, we identified licoflavone B, a natural flavonoid derived from licorice (*Glycyrrhiza* spp.), as a potent inhibitor of diverse influenza viruses, including multiple influenza A subtypes and type B virus. Mechanistic studies revealed that licoflavone B selectively targets the viral RNA-dependent RNA polymerase (RdRp), effectively suppressing viral replication. The compound exhibits a favorable selectivity index (SI = 14.9–29.9), indicating a promising therapeutic window. Molecular docking simulations identified potential binding interactions between licoflavone B and regions of the RdRp complex, which were further validated by dose-dependent inhibition of viral nucleoprotein (NP) and polymerase subunit PB2 expression in Western blot and immunofluorescence assays. In addition, licoflavone B maintained broad-spectrum antiviral activity against multiple influenza strains, including H1N1 (A/Puerto Rico/8/34), H3N2 (A/Darwin/9/2021), and a clinical influenza B isolate (B/Beijing/ZYY-B18/2018). These findings position licoflavone B as a promising lead compound for developing next-generation, broad-spectrum antiviral therapies against influenza and potentially other viruses.

## 1. Introduction

Influenza is an acute respiratory infectious disease, and influenza A has caused several global pandemics [1]. The unpredictable transmissibility and virulence profiles of emerging influenza viruses threaten public health and socioeconomic stability [2,3]. Three influenza pandemics emerged during the 20th century: 1918 H1N1 (Spanish flu), 1957 H2N2 (Asian flu), and 1968 H3N2 (Hong Kong flu). The 1918 influenza pandemic (Spanish Flu), the first well-documented outbreak of the influenza A virus (IAV), caused approximately 40 million fatalities worldwide [4]. Subsequently, the emergence of an influenza A (H1N1pdm09) variant in Mexico in April 2009 precipitated a 16-month global pandemic, which posed extensive negative impacts to international public health [5,6]. The development of novel broad-spectrum anti-influenza therapeutics is thus an urgent global health priority that requires immediate attention.

IAV is an enveloped virus and belongs to the Orthomyxoviridae family, with a segmented, negative-sense, single-stranded RNA genome [7]. Eight distinct genomic RNA segments, encapsulated within a lipid bilayer envelope, collectively encode at least 12 functional proteins [1], including receptor-binding hemagglutinin (HA), neuraminidase (NA), nucleoprotein (NP), and viral RNA-dependent RNA polymerase (RdRp) with three subunits—acidic polymerase (PA), basic polymerase 1 (PB1), and basic polymerase 2 (PB2) [2]. The RdRp complex consists of PB1, PB2, and PA proteins and is primarily responsible for viral RNA replication and transcription [8,9]. The NP and RdRp subunits are critical vRNA-associated proteins that bind to genomic segments [9].

Four principal classes of antiviral agents are clinically available for influenza management—M2 proton channel inhibitors (e.g., amantadine and rimantadine), NA inhibitors (including oseltamivir, peramivir, zanamivir, and laninamivir), the RdRp inhibitor favipiravir, and the cap-dependent endonuclease inhibitor baloxavir [10,11,12,13,14,15,16,17,18]. However, the emergence of widespread resistance to M2 inhibitors constitutes a significant therapeutic challenge, with more than 95% of circulating influenza A strains harboring the AM2-S31N mutation, conferring near-universal resistance to amantadine derivatives [11,19]. Global surveillance indicates that resistance rates for H1N1pdm09, H3N2, and influenza B viruses consistently remain below 1%. Nevertheless, the periodic identification of resistant variants underscores the critical need for sustained monitoring [20,21,22].

Existing RdRp inhibitor favipiravir is associated with adverse effects, and another RdRp inhibitor baloxavir can induce specific mutations (I38T or I38M) within the PA subunit of influenza viruses [15,23,24,25,26]. These substitutions markedly diminish the drug’s therapeutic efficacy, and corresponding resistant variants have been detected in circulation. These patterns necessitate the urgent development of novel broad-spectrum antivirals that target the evolutionarily conserved components of the viral replication cycle.

Traditional Chinese medicine (TCM) has demonstrated efficacy against influenza; however, its rich repository of bioactive constituents remains largely unexplored for their antiviral potential. In this study, we identified licoflavone B, a natural compound derived from licorice (*Glycyrrhiza* spp.), which has significant inhibitory activity against IAV replication. Although prior research on the anti-influenza effects of licoflavone B is limited, the plant from which it originates, licorice, and its extracts have been documented with the inhibition of SARS-CoV-2 [27]. Licorice has also been included as a drug for treating influenza in the influenza diagnosis and treatment plan released by the National Health Commission of China [28].

Licoflavone B also has anti-inflammatory and tumor-suppressive effects [29,30]. Furthermore, licoflavone B exhibits activity against Schistosoma species [31]. Through detailed mechanistic investigations, we found that this compound exerts its antiviral effects primarily via inhibition of viral RdRp. Our findings highlight licoflavone B as a promising candidate for novel influenza A therapeutics and contribute to the elucidation of the molecular basis underlying the empirical efficacy of TCM against influenza infections.

## 2. Materials and Methods

### 2.1. Compounds and Medicine

The compound and medicine used in this study were procured from Topscience (Shanghai, China), reconstituted in dimethyl sulfoxide (DMSO) to prepare 200 mM stock solutions, and then diluted in serum-free Dulbecco’s Modified Eagle Medium (DMEM) into working solutions of different concentrations. The final DMSO concentration was about 0.5%. DMEM was acquired from Gibco (Thermo Fisher Scientific, Waltham, MA, USA; Product No. C11960500BT), and it was added with 2 μg/mL L-1-tosylamido-2-phenylethyl chloromethyl ketone (TPCK) trypsin in all the virus relevant assays. The compound, medicine, and virus stock were all diluted using this medium.

### 2.2. Cells and Viruses

In this study, Madin–Darby canine kidney (MDCK) cells (CCL-34, ATCC) were engineered to incorporate a luciferase reporter gene [32,33,34], i.e., MDCK-Gluc cells. This system effectively induced reporter gene expression either through direct provision of influenza viral polymerase or indirectly via RdRP supplied by viral infection, showing compatibility with various influenza virus subtypes [35]. MDCK-Gluc cells were cultured in DMEM supplemented with 10% fetal bovine serum (FBS) and 1% penicillin-streptomycin (10,000 U/mL) in a 37 °C, 5% CO_2_ incubator. The parental cell line was obtained from the American Type Culture Collection and subsequently genetically modified (transformed) and cryopreserved in our laboratory.

Influenza virus strains, including influenza A/Puerto Rico/8/1934 (H1N1), and an influenza B virus B/Beijing/ZYY-B18/2018 (obtained from hospitalized patients in China, belonging to Yamagata subtypes), were propagated and archived within our laboratory facilities at the Institute of Microbial Epidemiology, Military Medical College. The influenza A/Darwin/9/2021 (H3N2) strain was provided by Zhejiang Provincial Center for Disease Control and Prevention.

### 2.3. Virus Inhibition Assay

The antiviral efficacy of the compounds was evaluated by quantifying the inhibition of viral luciferase expression. MDCK-Gluc cells (2.5 × 10^4^ cells/well) were seeded in 96-well plates at 37 °C in a 5% CO_2_ incubator for 24 h. Then, MDCK-Gluc cells were co-treated with serial dilutions of licoflavone B (0–200 μM) and influenza A/Puerto Rico/8/1934 (H1N1) [multiplicity of infection (MOI) = 0.1] at the same time; three duplicate wells were set up for each dilution concentration. Luminescence was measured using a microplate luminometer (Promega, Madison, WI, USA) at 24 h post-infection (hpi) (data acquired at 48 and 72 hpi were also collected in broad-spectrum antiviral potency tests with the strain A/Darwin/9/2021 (H3N2) and B/Beijing/ZYY-B18/2018 adopted in). Dose–response curves were generated, and half-maximal inhibitory concentration (IC_50_) values were calculated via GraphPad Prism 9.5 (GraphPad Software, Inc., San Diego, CA, USA).

### 2.4. Cell Viability Test

To evaluate the cytotoxicity profile of licoflavone B, MDCK-Gluc cells (2.5 × 10^4^ cells/well) were seeded in 96-well plates at 37 °C in a 5% CO_2_ incubator for 24 h. Then the cells were incubated with serially diluted concentrations of licoflavone B (0–200 μM) for 24 h, every dilution concentration was also set with three duplicate wells. Cell viability was quantified using a CCK-8 assay kit (Dojindo Laboratories, Kumamoto, Japan). Absorbance was recorded at 450 nm by a Tecan Sunrise microplate reader (Tecan Group Ltd., Männedorf, Switzerland). Data were collected and analyzed using GraphPad Prism 9.5 to calculate the half-maximal cytotoxic concentration (CC_50_). The selectivity index (SI), defined as the ratio of CC_50_ to IC_50_, assisting in testing the safety of the compound and, to a certain extent, evaluating its therapeutic window, i.e., the higher the SI value, the broader the therapeutic window [27].

### 2.5. Detection of Licoflavone B’s Inhibition Activity at the Late Stage of Viral Replication

The virus inhibition assay was conducted with the compounds added at 0 h of virus replication, based on our optimized viral inhibition assay protocol, as follows: MDCK-Gluc cells (2.5 × 10^4^ cells/well) were seeded in 96-well plates at 37 °C in a 5% CO_2_ incubator for 24 h. Then, MDCK-Gluc cells were infected with influenza A/Puerto Rico/8/1934 (H1N1; MOI = 0.1), and the cells were incubated with licoflavone B (0–200 μM) at the terminal stage of virus replication, i.e., the 6 h after virus addition. Each dilution concentration was tested in three duplicate wells. If licoflavone B still showed inhibitory activity, it indicated that the compound inhibits virus replication at both early and late stages, which would suggest that its scope of application and time period would be broader. Viral replication was quantified at 18 hpi by measuring luciferase activity. IC_50_ was determined using an established analytical method using GraphPad Prism 9.5.

### 2.6. Validation of Licoflavone B Suppression of Viral RdRp Activity Via Transient Transfection Assay

A total of 100 ng of the RNP expression plasmids derived from the PR8 virus (100 ng each of pHW2000-PB2, pHW2000-PB1, pHW2000-PA, and pHW2000-NP) was co-transfected into 80% confluence MDCK-Gluc cells seeded in 96-well plates at a density of 2.5 × 10^4^ cells per well using 1 µL of lipofectamine 3000. These plasmids encoded the full-length genomic sequences of influenza A/Puerto Rico/8/1934 (H1N1) RNP subunits cloned into the pHW2000 vector [35]. Six hours post-transfection, the MDCK-Gluc cells were incubated with licoflavone B or favipiravir (positive control) or oseltamivir (negative control). All drugs were diluted to concentrations between 0 and 200 μM and tested in three replicates each. Eighteen hours after drug exposure, viral RdRp expression was assessed by measuring luciferase expression using a microplate luminometer (Promega). The inhibition activity against the expression of RdRp of every drug was analyzed using GraphPad Prism 9.5.

### 2.7. Western Blotting

MDCK-Gluc cells (2.5 × 10^5^ cells/well) were seeded in 12-well plates at 37 °C in a 5% CO_2_ incubator for 24 h. Then, MDCK-Gluc cells were infected with influenza A/Puerto Rico/8/1934 (H1N1; MOI = 0.1), and the cells were incubated with licoflavone B at concentrations between 0 and 50 μM at the same time, with a concentration of 0 μM as the negative control (NC) and a concentration of 50 μM favipiravir as the positive control. The cells were lysed at 24 hpi. Protein lysates were prepared by RIPA buffer (Thermo Scientific, 89900) with a 100× protease inhibitor cocktail (Mei5 Biotechnology, Emeryville, CA, USA, MF182-plus-10). Viral NP and PB2 expression was used to evaluate the inhibitory effect of the compound against influenza A/Puerto Rico/8/1934 (H1N1). Following cell lysis, the samples were centrifuged at 20,000× *g* and 4 °C for 15 min, and then, the supernatants were collected. The lysates were resolved by SDS-PAGE gels and electrophoretically transferred onto nitrocellulose membranes (GE Healthcare, Chicago, IL, USA, 10600002). The membranes were blocked with 5% bovine serum albumin BSA in PBST solution for 1 h at room temperature. Then, the membranes were incubated with primary antibodies (Gentex, Zeeland, MI, USA, GTX636318 and GTX637893) overnight at 4 °C. After washing, the membranes were probed with horseradish peroxidase (HRP)-conjugated secondary antibodies for 1 h at room temperature. The immunoreactive bands were detected by a chemiluminescent HRP substrate (Millipore, Burlington, MA, USA, WBKLS0100) and visualized using a digital imaging system. Glyceraldehyde-3-phosphate dehydrogenase was used as the loading control. Gray scale analysis was performed using the ImageJ software (v1.8.0, NIH. Bethesda, MD, USA). Each target protein was detected in three independent, repeated experiments. Overall trends in the gray scale were visualized as bar charts using GraphPad Prism 9.5.

### 2.8. Immunofluorescence Assay

MDCK-Gluc cells (2.5 × 10^5^ cells/well) were seeded in 12-well plates at 37 °C in a 5% CO_2_ incubator for 24 h. Then, MDCK-Gluc cells were infected with influenza A/Puerto Rico/8/1934 (H1N1; MOI = 0.1), and the cells were incubated with licoflavone B at concentration between 0 and 25 μM at the same time, with 0 μM dilution as the NC and 50 μM favipiravir as the positive control. The supernatants were subsequently removed 24 h later, and the cells were fixed by 4% paraformaldehyde for 30 min at room temperature. Then, the cells were permeabilized using 0.2% Triton X-100 in PBST (15 min) after three washes with PBST. Nonspecific binding was blocked with 2% FBS in PBST for 1 h at room temperature. Primary immunolabeling was performed overnight at 4 °C with murine anti-influenza NP or PB2 antibodies. Then, the cells were incubated with FITC-conjugated goat anti-mouse immunoglobulin G secondary antibody (Abcam, Cambridge, UK, ab6717) for 1 h after three washes by PBST. Nuclear counterstaining was performed using 4′,6-diamidino-2-phenylindole (Abcam, ab104139) for 15 min. Fluorescent images were acquired using a high-content imaging system (Cytation 1; BioTek, Winooski, VT, USA). The green fluorescent protein (GFP) level was calculated using ImageJ software (v1.8.0, NIH). Three perspectives in each figure were selected to summarize the GFP level at every drug concentration. The overall trends were visualized in a bar chart using GraphPad Prism 9.5.

### 2.9. Quantitative Real-Time RT-PCR

To quantify intracellular viral RNA, total RNA of influenza was isolated using the RNeasy Mini Kit (Takara Bio, Tokyo, Japan; RR064A). MDCK-Gluc cells (2.5 × 10^4^ cells/well) were seeded in 96-well plates at 37 °C in a 5% CO_2_ incubator for 24 h. Then, MDCK-Gluc cells were co-treated with serial dilutions of licoflavone B (0–200 μM) and influenza virus (MOI = 0.1) for 24 h, and the supernatant was collected to test the viral RNA load. The primers used for detection are shown in Table 1. All reactions were performed in three duplicate for each concentration with appropriate controls. The Ct values for each reaction were normalized against that of the NC group. Trends were visualized in bar charts using GraphPad Prism 9.5 after the normalization.

### 2.10. Computerized Virtual Docking Licoflavone B with RdRp

Computational virtual docking was performed to investigate the inhibitory potential of licoflavone B on RdRp. Owing to the absence of a resolved crystal structure for the target strain, homology modeling was performed using the reference sequences of the influenza A/Puerto Rico/8/1934 (H1N1) obtained from the National Library of Medicine [NCBI; www.ncbi.nlm.nih.gov (accessed on 20 June 2025)]. Since the precise crystal structures of these specific viral RdRps remain undetermined, homology models were first generated using SWISS-MODEL [https://swissmodel.expasy.org/ (accessed on 20 June 2025)] based on their respective amino acid sequences. We selected the structure with the highest sequence similarity of A/Puerto Rico/8/1934 (H1N1) for molecular docking. The homology modeling structure used for A/Puerto Rico/8/1934 (H1N1) was the template 6RR7 [36], with 96.68% sequence identity. Molecular docking simulations were conducted using Discovery Studio 2016 to analyze the interactions between RdRp and licoflavone B. A reliable analysis of the 64 putative active RdRp pockets was conducted. Each pocket was systematically docked with licoflavone B, and successfully docked complexes were evaluated based on their binding affinity scores. The highest-scoring binding sites were subjected to detailed binding-site analysis and visualization using LigPlot+ v.2.2.8 and ChimeraX 1.9.

## 3. Results

### 3.1. Inhibitory Effect and Cytotoxicity of Licoflavone B

**Figure 1:**
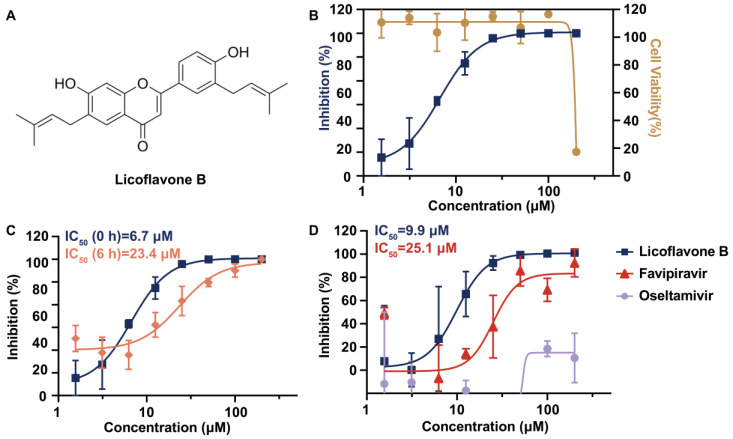
Structural characterization and inhibitory effect of licoflavone B on influenza viruses. (**A**) Chemical structure of licoflavone B. (**B**) Inhibitory effect of licoflavone B on influenza A virus [A/Puerto Rico/8/34 (H1N1)] and its cytotoxicity. (**C**) Inhibitory activity against influenza A virus [A/Puerto Rico/8/34 (H1N1)] when licoflavone B was added at 0 and 6 hpi. (**D**) Validation of the inhibitory effect of licoflavone B on RNA-dependent RNA polymerase expression using a four-plasmid cotransformation system assessed using a luciferase reporter gene assay. Half-maximal inhibitory concentration (IC_50_) values are shown in the figures, unless they could not be determined.

We evaluated the antiviral activity of licoflavone B (Figure 1A) against A/Puerto Rico/8/34 (H1N1), a historic reference strain widely used in in vitro studies owing to its high yield and low pathogenicity. The assay demonstrated significant antiviral activity of licoflavone B against the influenza virus, with an IC_50_ of 6.7 μM (Figure 1B). Cytotoxicity assessments were performed to determine the CC_50_ to exclude cytotoxicity-mediated false positives. The CC_50_ of licoflavone B was between 100 and 200 μM (Figure 1B), indicating detectable cytotoxicity only at the highest tested concentrations. The SI, calculated as the CC_50_/IC_50_ ration, was between 14.9 and 29.9, indicating great therapeutic specificity and cellular safety to a certain extent, confirming the antiviral efficacy of licoflavone B against IAV.

### 3.2. Verification of the Inhibitory Activity of Licoflavone B at Late Stage of Virus Replication

To study the mechanisms underlying the anti-influenza activity of licoflavone B, an assay was performed to verify the inhibitory activity of licoflavone B at different stages of virus replication. MDCK-Gluc cells infected with A/Puerto Rico/8/34 (H1N1) were treated with a predetermined effective concentration of licoflavone B at 6 hpi, and the viral load was quantified 18 h later. The results indicated that the compound effectively suppressed viral replication when administered within the compound given at 6 hpi with an IC_50_ of 24.7 μM (Figure 1C). Although the observed IC_50_ value was relatively higher, which may be attributed to the advanced stage of viral replication with substantial viral particles already released at the time of treatment, the compound still maintained antiviral efficacy. This persistence of antiviral efficacy suggests interference with a process that occurs during viral replication. Therefore, we hypothesized that licoflavone B has inhibition activity at both the early stage and the late stage of viral replication.

### 3.3. Validation of the Viral Target

A luciferase reporter gene assay was performed to quantitatively assess the viral RdRp activity. This system reconstituted the influenza viral transcription–replication machinery through the co-transfection of MDCK-Gluc cells with plasmids encoding RNP components (NP, PA, PB1, and PB2) from the A/Puerto Rico/8/34 (H1N1) strain. At 6 h after transfection, the cells were treated with licoflavone B, favipiravir, or oseltamivir (0–200 μM). Luciferase activity was quantified 18 h after co-transfection. Licoflavone B suppressed polymerase activity in a concentration-dependent manner, with an IC_50_ of 9.9 μM (Figure 1D). This inhibitory potency was greater than that of the reference RdRp inhibitor, favipiravir (IC_50_ = 25.1 μM; Figure 1D). In contrast, the NA inhibitor, oseltamivir, had no significant effect on polymerase function (Figure 1D). These differential inhibitory profiles suggest that licoflavone B specifically targets the IAV RdRp.

### 3.4. Broad-Spectrum Antiviral Potency

Next, we evaluated the broad-spectrum inhibitory potential of licoflavone B against various influenza viruses, including contemporary clinical isolates. The viral panel included (1) the historic reference strain A/Puerto Rico/8/34 (H1N1); (2) the contemporary seasonal strain A/Darwin/9/2021 (H3N2); and (3) a clinical influenza B virus B/Beijing/ZYY-B18/2018 strain obtained from hospitalized patients, belonging to Yamagata subtypes. The antiviral efficacy against influenza A strains was assessed using a luciferase reporter gene assay. However, this assay cannot be applied for testing for influenza B virus. RT-qPCR was used for all strains to directly quantify the viral load. The inhibitory effect was recorded at 24, 48, and 72 hpi to test the compounds’ effect, with 72 hpi set as the terminal state.

**Figure 2:**
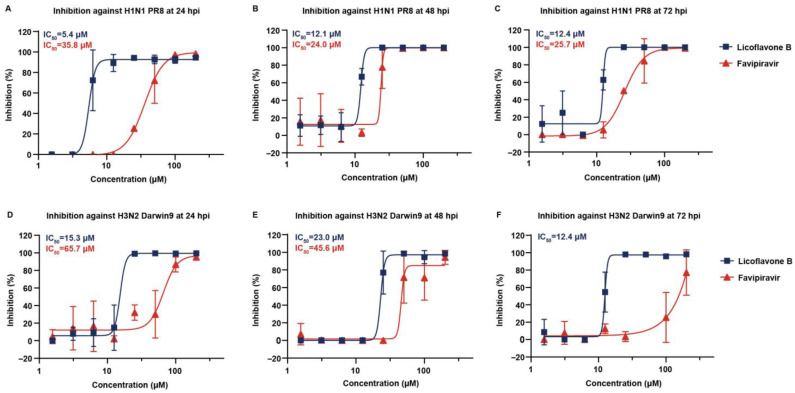
Inhibitory effect of licoflavone B on different viruses at different time points using the luciferase reporter gene system. Validation of the inhibitory effect of licoflavone B on influenza A virus [A/Puerto Rico/8/34 (H1N1)] at (**A**) 24, (**B**) 48, and (**C**) 72 hpi. Validation of the inhibitory effect of licoflavone B on Influenza A virus [A/Darwin/9/2021 (H3N2)] at (**D**) 24, (**E**) 48, and (**F**) 72 hpi. Half-maximal inhibitory concentration (IC_50_) values are shown unless they could not be determined.

The results showed that licoflavone B had a stable inhibitory effect against A/Puerto Rico/8/34 (H1N1) at 24, 48, and 72 hpi, with IC_50_ values of 5.4, 12.1, and 12.4 μM, respectively, which were lower to that of the positive control favipiravir (Figure 2A–C). In addition, licoflavone B showed advanced inhibitory effects against A/Darwin/9/2021 (H3N2), with IC_50_ values of 15.3, 23.0, and 12.4 μM at 24, 48, and 72 hpi, respectively, whereas favipiravir had an effect at 24 and 48 hpi and had no significant effect at 72 hpi (Figure 2D–F). These preliminary results suggest that licoflavone B has broad-spectrum inhibitory effects against influenza viruses. The results showed that licoflavone B had the best inhibitory activity against A/Puerto Rico/8/34 (H1N1), followed by A/Darwin/9/2021 (H3N2). The inhibition activity of licoflavone B against the H1N1 strain was superior to that against the H3N2 strain generally. These inhibitory differences are likely due to the sequence- and structure-related variations in H1N1 and H3N2. Further strains should be tested to further verify the broad-spectrum antiviral potency of licoflavone B and provide a more reliable assessment of its antiviral spectrum.

**Figure 3:**
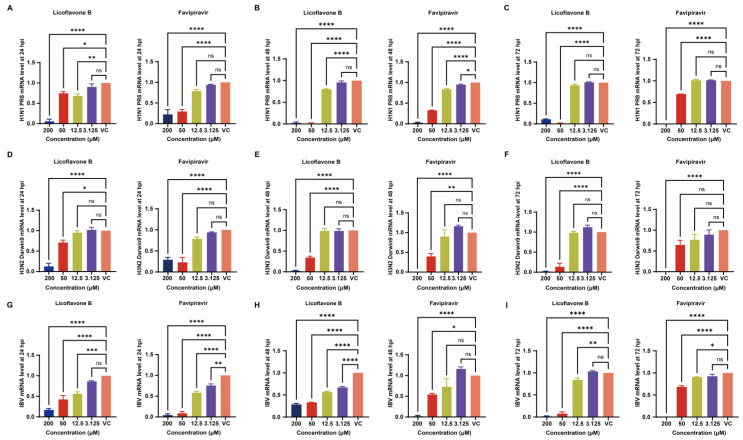
Inhibitory effect of licoflavone B on different viruses at different time points using real-time quantification. Validation of the inhibitory effect of licoflavone B on influenza A virus [A/Puerto Rico/8/34 (H1N1)] at (**A**) 24, (**B**) 48, and (**C**) 72 hpi; validation of the inhibitory effect of licoflavone B on influenza A virus [A/Darwin/9/2021 (H3N2)] at (**D**) 24, (**E**) 48, and (**F**) 72 hpi. Validation of the inhibitory effect of licoflavone B on influenza B virus B/Beijing/ZYY-B18/2018 at (**G**) 24, (**H**) 48, and (**I**) 72 hpi. ns *p* > 0.05, * *p* < 0.05, ** *p* < 0.01, *** *p* < 0.001 and **** *p* < 0.0001.

These findings were further corroborated by the results of the RT-qPCR assay for influenza virus detection. RT-qPCR analysis revealed significant dose-dependent inhibition by licoflavone B in all tested strains. Licoflavone B exhibited a remarkable effect against A/Puerto Rico/8/34 (H1N1), and the effect surpassed that of favipiravir at 48 and 72 hpi (Figure 3A–C). In addition, licoflavone B had a significant effect on A/Darwin/9/2021 (H3N2), showing stronger inhibition than favipiravir at 72 hpi (Figure 3D–F). Nevertheless, licoflavone B could inhibit influenza B virus, and its effect was stronger than that of favipiravir at 72 hpi (Figure 3G–I).

### 3.5. Viral Protein Expression Analysis Demonstrates Dose Dependent Inhibition by Licoflavone B

To validate our findings, we evaluated viral protein expression in IAV-infected cells using Western blotting and immunofluorescence. MDCK-Gluc cells infected with A/Puerto Rico/8/34 (H1N1; MOI = 0.1) were treated with licoflavone B to assess its effects on viral protein synthesis and subcellular distribution of viral proteins. The NP and PB2 proteins were selected as target antigens because of their integral roles in the viral RNP complex.

**Figure 4:**
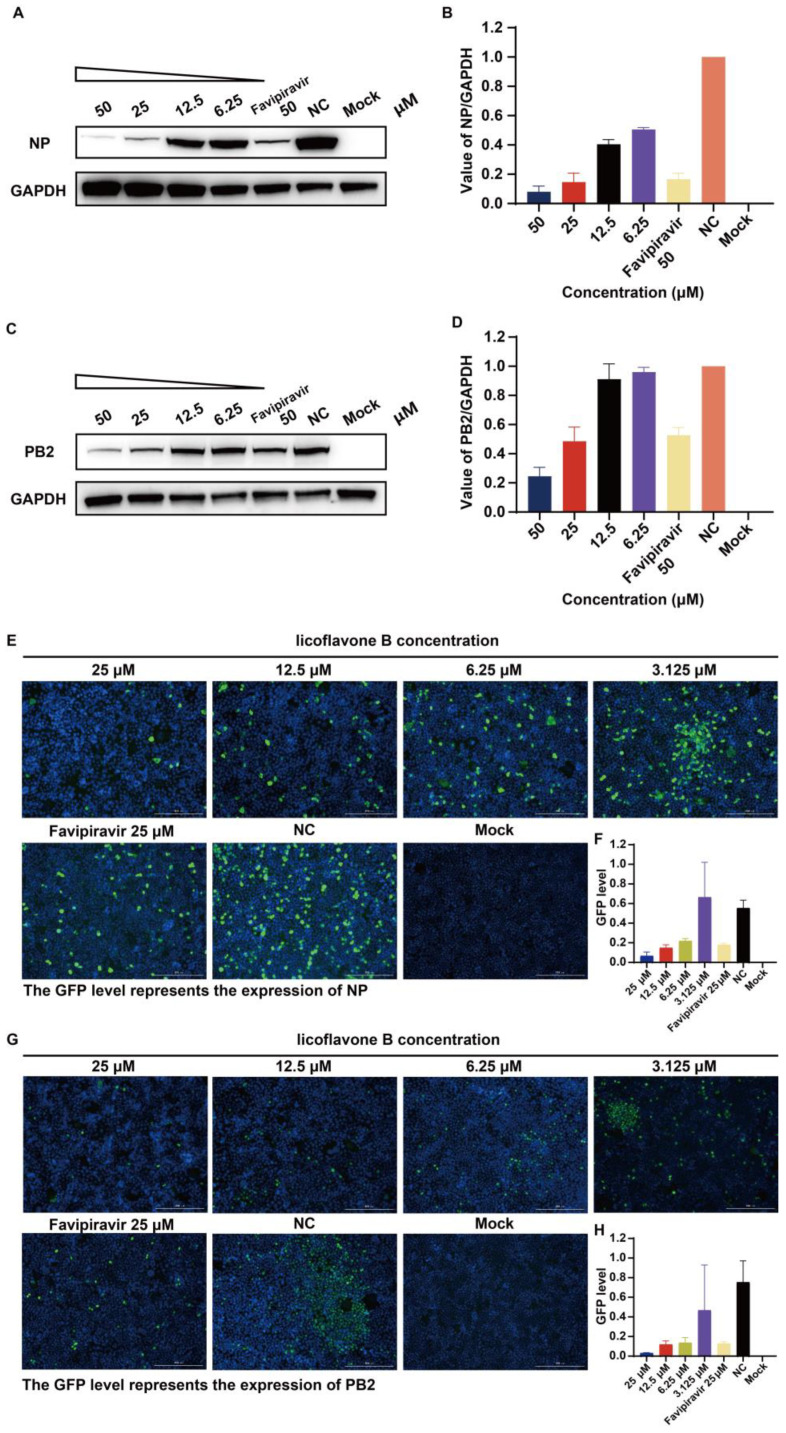
Verification of the inhibitory effect of licoflavone B on influenza A virus using Western blotting and immunofluorescence assay. (**A**) Expression of influenza A nucleoprotein (NP) was characterized by Western blotting, and bands were quantified (**B**) using ImageJ. (**C**) Expression of influenza A basic polymerase 2 (PB2) was characterized by Western blotting, and bands were quantified (**D**) using ImageJ. (**E**) The dose-dependent inhibitory effect of licoflavone B on influenza A virus [A/Puerto Rico/8/34 (H1N1)] was validated using immunofluorescence to quantify viral nucleoprotein (NP), and the expression of NP was evaluated by calculating the level of GFP (**F**) using ImageJ. Scale bar: 200 µm (**G**) The dose-dependent inhibitory effect of licoflavone B on influenza A virus [A/Puerto Rico/8/34 (H1N1)] was validated using immunofluorescence to quantify viral basic polymerase 2 (PB2), and the expression of PB2 was evaluated by calculating the level of GFP (**H**) using ImageJ, Scale bar: 200 µm.

Western blot analysis revealed dose-dependent suppression of both viral proteins in infected cells following licoflavone B treatment (Figure 4A,C). Gray scale values for Western blot bands were statistically analyzed based on data obtained from three independent replicates (Figure 4B,D). Concomitant immunofluorescence analysis revealed a dose-dependent reduction in viral antigen expression (Figure 4E,F). Quantitative image analysis further indicated that licoflavone B exerted a significantly greater inhibitory potency against viral protein expression than the reference compound favipiravir at equivalent concentrations (Figure 4G,H), corroborating the superior antiviral efficacy of licoflavone B observed in the previous functional assays.

### 3.6. Molecular Docking

To elucidate the structural basis of RdRp inhibition and characterize the potential binding interactions between licoflavone B and IAV RdRp, computational molecular docking was performed using a high-resolution crystal structure.

**Figure 5:**
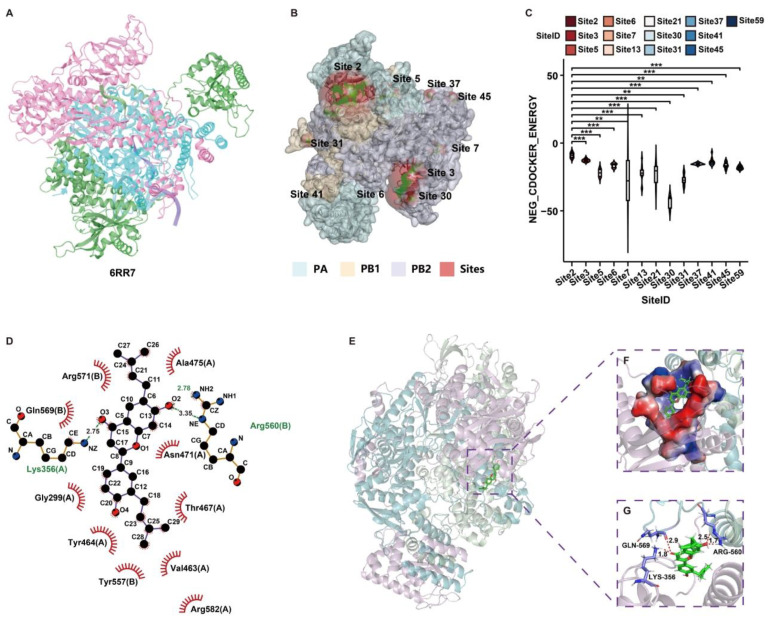
Computational identification of binding sites for licoflavone B inhibition in H1N1 RNA-dependent RNA polymerase (RdRp) homology models. (**A**) Structure of RdRp of 6RR7 for influenza virus A/Puerto Rico/8/34 (H1N1) using homologous modeling. (**B**) Active sites of 6RR7 that can successfully dock with licoflavone B (active sites fully occluded by structural elements are not annotated in the figure). (**C**) CDOCKER_ENERGY assessment of the activity pockets of licoflavone B. ** *p* < 0.01, *** *p* < 0.001. Optimal docking conformations of licoflavone B scored by docking are shown in (**D**) 2D and (**E**) 3D diagrams. (**F**) Solid surface mode in the binding site of licoflavone B. (**G**) Hydrogen bond interactions (red dash) between licoflavone B and the backbone residues of the polymerase.

Given the demonstrated inhibitory activity of licoflavone B against A/Puerto Rico/8/34 (H1N1), we conducted molecular docking studies targeting their RdRp domains. The RdRp structures of A/Puerto Rico/8/34 (H1N1) were modeled on template 6RR7 [36] (96.68% sequence identity; Figure 5A).

A reliable structural analysis of the heterotrimeric RdRp complex performed using Discovery Studio 2016 identified 64 ligand-binding pockets within the homology models of 6RR7. Systematic molecular docking simulations were conducted against all identified pockets to determine the potential binding sites. The docking results indicated favorable binding energetics for licoflavone B within the polymerase complex, suggesting a significant molecular interaction potential. Specifically, licoflavone B yielded productive docking poses in 20 of the 64 pockets analyzed in the 6RR7 model (Figure 5B). Based on the calculated binding affinities, the highest-scoring pocket demonstrating robust binding potential was selected for further characterization. Comparative binding energy analysis revealed that Site 2 of 6RR7, located within the PA subunit, consistently exhibited superior docking scores (Figure 5C), indicating that it might be the most thermodynamically favorable binding sites. Molecular interactions within Site 2 of 6RR7 was characterized through high-resolution structural visualization and interaction analysis using LigPlot+ v.2.2.8 and ChimeraX 1.9 (Figure 5D–G), enabling a detailed examination of the binding conformations and specific protein–ligand contacts. These analyses identified critical amino acid residues within Site 2 of 6RR7 that mediate key interactions with licoflavone B, demonstrating a stable binding affinity for RdRp.

## 4. Discussion

In this study, we demonstrate that licoflavone B, a flavonoid isolated from licorice (*Glycyrrhiza* spp.), is a potent inhibitor of influenza A virus replication. Licoflavone B is a kind of small-molecule compound, which can usually easily cross the biological barriers and enters the interior of cells to exert their effects. Moreover, such drugs have low immunogenicity, and it is easy to optimize and modify their chemical structure. Our study showed that licoflavone B has advanced influenza inhibition activity and cytotoxicity at high dose, implying its use as a new antiviral small-molecule drug candidate and the necessity of modifying its structure and weakening its cytotoxicity. Future studies should detect toxic and protective effects of licoflavone B in vivo and collect pharmacokinetics data.

In this study, we innovatively adopted a new model to test the influenza inhibition activity of licoflavone B. The antiviral mechanism was pinpointed with a universal MDCK-based VIRG (virus-induced reporter gene) system [35]. Briefly, MDCK cells were engineered to stably express Gaussian luciferase (Gluc) under the control of an RNA polymerase II promoter flanked by influenza A virus RNA promoters. Upon infection, the viral RdRp complex recognizes these viral promoters and drives Gluc transcription and subsequent secretion of luciferase, so that Gluc activity in the culture supernatant rises in direct proportion to viral RdRp activity. Using this platform, we show that licoflavone B selectively suppressed the viral RdRp complex—the central enzymatic machinery governing viral RNA replication and transcription—thereby blocking propagation of divergent influenza A subtypes. Initial screening identified licoflavone B as a potent inhibitor of influenza A/Puerto Rico/8/1934 (H1N1), with an IC_50_ of 6.7 μM. The SI of licoflavone B was between 14.9 and 29.9, providing preliminary validation of the inhibitory efficacy and safety profile of licoflavone B. The SI value represents the therapeutic potential of a drug. To a certain extent, the higher the SI value, the stronger the antiviral efficacy and the safety of the drug. In general, an SI value ≥ 10 identifies a compound that is worthy of further investigation [37,38]. The SI value of licoflavone B ensured its antiviral activity, yet it still illustrated that licoflavone B has cytotoxicity at high dosage. An ideal antiviral drug would be cytotoxic only at very high concentrations and have antiviral activity at low concentrations, eliminating the target virus at concentrations well below its cytotoxic concentration. Therefore, it is necessary to modify the structure of licoflavone B, reducing its cytotoxicity under the premise of retaining its antiviral activity in future study. Additionally, we only assessed the CC_50_ at 24 hpi. It will be of great significance to evaluate more time points to test the difference of the value of CC_50_, since we observe the inhibition activity of licoflavone B at multiple time points. It will be helpful for us to comprehensively evaluate the therapeutic window of licoflavone B.

We hypothesized that the licoflavone B could exert inhibitory effects at both the early stage and the late stage of viral replication, since the inhibition activity of licoflavone B against the virus still exists at both 0 hpi and 6 hpi. Then, the luciferase reporter gene system was used to indicate that licoflavone B targets the viral RdRp complex following the co-transfection of RNP expression plasmids derived from the PR8 virus (pHW2000-PB2, pHW2000-PB1, pHW2000-PA, and pHW2000-NP). The results showed that the inhibition of RdRp expression by licoflavone B was stronger than that by RdRp inhibitor favipiravir, which was added to the influenza diagnosis and treatment plan issued by the National Health Commission of China in 2025 [28]. Favipiravir is applicable in the treatment of novel or re-prevalent influenza in adults. Notably, favipiravir is only permitted for use only in Japan and China with relatively limited scope for clinical medication. Additionally, a previous study demonstrated that licoflavone B targeted the NA of influenza viruses [39]. Our study first reveals the possibility that the target of licoflavone B lies in the RdRp of influenza viruses. This discrepancy has prompted renewed contemplation. NA inhibitors exert their effects extracellularly by blocking viral egress during the late stage of viral replication, while RdRp inhibitors act intracellularly to disrupt viral replication processes, primarily functioning during the early replication phase. Our experimental data reveal that licoflavone B exhibits inhibitory activity at both early and late stages of viral replication. Moreover, the IC_50_ value at the late stage demonstrates a certain degree of increase, which appears to weaken the likelihood of NA being its primary target. Therefore, further validation through ELLA and MUNANA assays is warranted [40], alongside observations at additional time points to monitor dynamic changes in its inhibitory activity [41].

A systematic assessment of the inhibitory efficacy of licoflavone B at multiple post-infection time points generally validated the broad-spectrum anti-influenza activity of the compound. The influenza virus strains utilized in this investigation comprised the historic reference strain A/Puerto Rico/8/34 (H1N1), contemporary seasonal strain A/Darwin/9/2021 (H3N2), and clinical strain influenza B virus B/Beijing/ZYY-B18/2018. Notably, both favipiravir and licoflavone B exhibited a similar, time-dependent efficacy reduction in IC_50_ values in our tests though it is on the same order of magnitude, especially at 24 hpi and 72 hpi. The results of the RT-qPCR assay indicated similar trends. We deduced that as the viruses continue to replicate over time, the effect of the drugs likely declined to a certain extent. However, the general inhibitory activity of licoflavone B was stronger than that of favipiravir, only slightly weakened throughout the extended incubation period, suggesting distinct mechanisms with potential therapeutic advantages. Collectively, our findings initially demonstrate that licoflavone B exhibits broad-spectrum anti-influenza activity, making it a promising candidate for the development of novel broad-spectrum antiviral therapeutics. In future studies, more types and subtypes of influenza virus should be tested to verify the broad-spectrum antiviral activity of licoflavone B, especially the influenza B virus. Also, the difference of the inhibition activity of licoflavone B against the contemporary strains and the historical strains need to be take into consideration, and more comparison of them should be conducted and analyzed. More reference influenza B strains should be brought into the tests, since the subtype of influenza B strain used in this study was a clinical isolated strain rather than other classic influenza B strains.

Another characteristic of this study was the multi-angle assays of proving the inhibition activity of licoflavone B. Besides the results obtained using the luciferase reporter gene system and RT-qPCR assay, Western blotting and immunofluorescence assays revealed reduced expression levels of both NP and PB2 in IAV-infected cells following licoflavone B treatment. These data visually indicate its dose-dependent inhibitory effect on the influenza virus. However, our study also had some limitations. For example, the test used to determine the impact of licoflavone B on the infectious virus yield was insufficient. In addition, mutagenesis studies of the active site calculated by the computer simulations need to be conducted to ensure that the residues at the active site indeed participate in the combination. We will further investigate these residues to facilitate the precise mapping of the antiviral interaction sites of licoflavone B. Also, performing only one independent experiment is another significant limitation of the study.

In summary, this study demonstrates that licoflavone B functions as a direct-acting RdRp inhibitor with potent, broad-spectrum anti-influenza activity. The source plant of licoflavone B, licorice, was included in the influenza diagnosis and treatment scheme and has been used to treat influenza and other respiratory infectious diseases related to influenza in China for more than two thousand years. Moreover, licorice extract has been shown to inhibit SARS-CoV-2 [27]. Our findings help elucidate the mechanisms underlying the effects of licorice in treating influenza. The inhibitory effect of licoflavone B on influenza virus further substantiate its potential as a promising lead compound for the development of novel broad-spectrum anti-influenza therapeutics.

## Figures and Tables

**Table 1:** Primers used in real-time qPCR.

	**Influenza A virus primers**
Forward	5′-GACCRATCCTGTCACCTCTGAC-3′
Reverse	5′- GGGCATTYTGGACAAAKCGTCTACG-3′
Probe	5′-[FAM]TGCAGTCCTCGCTCACTGGGCACG[BHQ-1]-3′
	**Influenza B virus primers**
Forward	5′-TCCTCAACTCACTCTTCGAGCG-3′
Reverse	5′-CGGTGCTCTTGACCAAATTGG-3′
Probe	5′-[FAM]CCAATTCGAGCAGCTGAAACTGCGGTG[BHQ-1]-3′

## Data Availability

The original contributions presented in this study are included in the article. Further inquiries can be directed to the corresponding authors.
